# A tale of two hemispheres: contrasting socioemotional dysfunction in right- versus left-lateralised semantic dementia

**DOI:** 10.1590/S1980-57642013DN70100014

**Published:** 2013

**Authors:** Muireann Irish, Fiona Kumfor, John R. Hodges, Olivier Piguet

**Affiliations:** 1 School of Psychology, University of New South Wales, Sydney, Australia. Neuroscience Research Australia, Sydney, Australia, and School of Medical Sciences, University of New South Wales, Sydney, Australia. PhD, Research Fellow at the School of Psychology, University of New South Wales, Sydney, Australia.; 2 Masters, PhD, Candidate at Neuroscience Research Australia, Randwick, Sydney, Australia.; 3 Professor and Senior Principal Research Fellow at Neuroscience Research Australia, Randwick, Sydney, Australia.; 4 Associate Professor and Senior Research Fellow at Neuroscience Research Australia, Randwick, Sydney, Australia.

**Keywords:** semantic dementia, emotion processing, frontotemporal dementia, hemispheric lateralisation

## Abstract

**OBJECTIVE:**

Semantic dementia, a subtype of frontotemporal lobar degeneration, is characterised by cross-modal loss of conceptual knowledge attributable to progressive degeneration of the left anterior temporal lobe. Much less is known regarding the clinical presentation of SD patients with predominantly right-lateralised atrophy. Recent reports emphasise marked socioemotional and behavioural disturbances in such cases. Given the importance of the right anterior temporal lobes in social cognition, we hypothesised that socioemotional functioning would be disproportionately affected in right versus left-lateralised SD cases.

**METHODS:**

We assessed well-characterised cases of predominantly right (n=10) and left (n=12) SD and 20 matched healthy controls on tests of emotion processing and interpersonal functioning.

**RESULTS:**

Right SD cases showed disproportionate difficulties in the recognition of positive and negative facial emotions, specifically happiness and anger, compared with left SD cases. Deficits in anger recognition persisted in right SD despite covarying for facial and semantic processing. On a contextually rich task of emotion recognition using multimodal videos, no subgroup differences were evident. Finally, empathic concern was rated as significantly lower by caregivers of right versus left SD cases. Overall, the extent of socioemotional disturbance was associated with the degree of behavioural changes in SD.

**CONCLUSION:**

Our results reveal considerable overlap in the extent to which socioemotional processes are disrupted in left and right-lateralised cases of SD. Notably, however, right SD cases show disproportionate deficits for recognition of facial emotions and the capacity for empathic concern, supporting a specialised role for the right anterior temporal lobes in mediating these cognitive functions.

## Abstract (pt)

**OBJETIVO:**

A demência semântica (DS), um subtipo de degeneração lobar frontotemporal, é caracterizada por perda multimodal do conhecimento conceitual atribuída à degeneração progressiva do região anterior do lobo temporal esquerdo. Sabe-se menos sobre o quadro clínico de pacientes com DS em que a atrofia é localizada predominantemente à direita. Relatos recentes têm enfatizado marcantes distúrbios socioemocionais e comportamentais em tais casos. Dada a importância da região anterior do lobo temporal direito na cognição social, aventamos a hipótese de que o funcionamento socioemocional seria desproporcionalmente afetado nos casos de DS com atrofia lateralizada à direita.

**MÉTODOS:**

Foram avaliados os desempenhos de casos bem caracterizados de DS com atrofia do lobo temporal predominantemente à direita (n=10) e à esquerda (n=12) e 20 controles saudáveis em testes de processamento de emoções e funcionamento interpessoal.

**RESULTADOS:**

Casos de DS com atrofia predominante à direita apresentaram dificuldades desproporcionadas no reconhecimento de emoções faciais positivas e negativas, especificamente expressões de felicidade e raiva, em comparação com os casos de atrofia à esquerda. Os déficits no reconhecimento de raiva persistiram depois de excluídas as covariações com processamento facial e semântico. Em uma tarefa contextualmente rica de reconhecimento de emoções através de vídeos multimodais, não houve diferenças entre os subgrupos. Por fim, preocupação empática foi classificada por cuidadores como significativamente menor nos casos com atrofia à direita. Em geral, o grau de perturbação socioemocional foi associado com o grau de alterações comportamentais na DS.

**CONCLUSÃO:**

Nossos resultados revelam uma considerável sobreposição na medida em que os processos socioemocionais são rompidos tanto em casos com atrofia predominante à direita como à esquerda. Notavelmente, entretanto, os casos com DS com atrofia predominante à direita apresentam déficits desproporcionais no reconhecimento de emoções faciais e na capacidade de preocupação empática, dando suporte à hipótese de um papel especializado das regiões anteriores do lobo temporal direito na mediação dessas funções cognitivas.

## INTRODUCTION

Semantic dementia (SD) is a clinical syndrome associated with focal degeneration of the anterior temporal lobes of the brain, manifesting in the progressive cross modal deterioration of general conceptual knowledge.^1,2^ Patients with SD present with severe semantic impairments due to asymmetrical, primarily left-sided, brain atrophy including the anterior and medial portions of the temporal lobe.^3,4^ Extensive clinical and anatomical characterisations of predominantly left-sided SD cases concord with the lateralisation of verbal skills and phonological representations to the left hemisphere^5^ and have proved particularly illuminating for our understanding of the complex cognitive architecture of the semantic and episodic memory systems of the brain.^6^ In contrast, however, a dearth of information exists regarding the less common presentation of SD with predominant right-lateralised atrophy.

To date, clinical data on right SD have been largely gleaned from individual or case series reports, the majority of which emphasise the presence of prosopagnosia, loss of empathy, behavioural disinhibition, and disruptions to interpersonal functioning.^7-11^ Recent group studies have revealed episodic memory and spatial navigation deficits,^12^ and alterations in food preferences^13^ in this group. Eccentric social behaviour, with alterations in dressing, personal hygiene, sociopathic behaviours, irritability, and impulsivity, appear more frequent in patients with predominantly right sided pathology in comparison with left-sided SD cases.^7^ Together with loss of empathy and insight, disinhibition, and difficulties in affect regulation, such alterations in social comportment may bias the clinician to misdiagnose right SD as behavioural variant frontotemporal dementia, particularly when structural neuroimaging is not available.^10^

The emergence of florid socioemotional and behavioural changes in the majority of right SD cases resonates well with the position that the right anterior temporal lobe plays a pivotal role in the abstraction of conceptual knowledge from the social domain.^14,15^ Despite recent group studies involving large samples of right SD cases^10,12^ much remains to be elucidated regarding the clinical features that may be specific to this syndrome. While the evidence to date suggests that socioemotional deficits may be particular to right SD,^8,13^ deficits in emotion processing are widely reported in left SD cases across a range of modalities including facial stimuli,^16,17^ musical excerpts,^18^ and emotional words.^19^ Importantly, while left SD patients do show marked impairments in emotion processing, such deficits appear attributable, in part, to the verbal demands of the tasks used.^17^ Further, changes in the capacity for empathic concern have been documented in left SD.^20^ These changes, which encompass both the cognitive and affective aspects of interpersonal functioning,^21,22^ correlate with the extent of atrophy in right, rather than left, temporal lobe structures.^20^

The extent to which social functioning is differentially compromised in right versus left lateralised cases of SD remains unknown. Current evidence suggests that emotion recognition and interpersonal functioning may be disproportionately affected in right SD compared to left SD. To our knowledge, however, group studies comparing socioemotional functioning in right versus left SD cases have not been conducted. The objective of this study was to investigate profiles of socioemotional dysfunction in a sample of predominantly right-lateralised cases of SD and to contrast their performance with a well-characterised cohort of age-, education- and disease-matched left-sided SD cases. We predicted that, right SD cases would show disproportionate deficits in comparison to the left-sided cases for those functions largely ascribed to the right anterior temporal lobes, namely emotion recognition, and the capacity for empathic concern.

## METHODS

**Figure 1:**
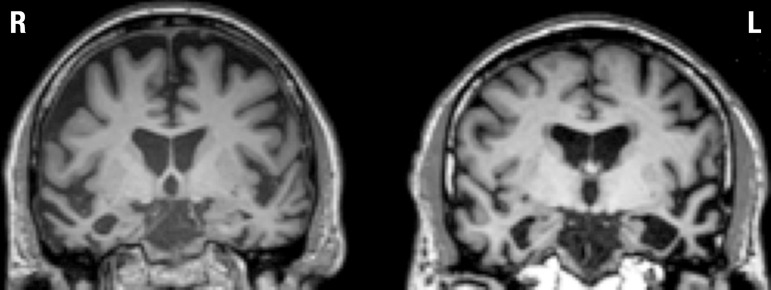
Structural T1-weighted coronal images of the representative patterns of atrophy in semantic dementia patients with predominantly right (R) or left (L) temporal lobe atrophy.

**Participants.** Twenty-two patients with semantic dementia (SD) and 20 older age- and education-matched healthy controls took part in this study. Diagnosis of SD cases was established in line with current clinical diagnostic criteria^23^ by consensus among a multidisciplinary team based on extensive clinical investigations, cognitive assessment, and review of structural neuroimaging scans. Briefly, individuals diagnosed with left SD exhibited progressive loss of word meaning manifesting in impaired naming and comprehension, in the context of relatively spared episodic memory, with neural atrophy predominantly in the left anterior temporal lobe. In contrast, patients diagnosed with right SD presented with loss of semantic knowledge, marked prosopagnosia, and behavioural changes, with evidence of extensive neural atrophy predominantly in the right temporal lobe on structural MRI. Figure 1 displays representative scans for right and left SD cases.

Controls scored 0 on the sum of boxes score of the Clinical Dementia Rating scale (CDR)^25^ and 88 or above on the Addenbrooke's Cognitive Examination-Revised (ACE-R).^25^ Exclusion criteria included prior history of mental illness, significant head injury, movement disorders, cerebrovascular disease, alcohol or substance abuse, and limited english proficiency. Ethical approval was obtained from the Southern Eastern Sydney and Illawarra Area Health Service and the University of New South Wales ethics committees. Informed consent was obtained from all participants, or their person responsible.

**General cognitive screening.** Participants completed a neuropsychological battery of tests to assess general cognitive functioning (ACE-R),^25^ visual episodic memory (Rey Complex Figure),^26^ semantic processing including an index of Naming and Comprehension,^27^ speed of processing (Trail Making Test Part A),^28^ verbal fluency,^29^ (FAS),^29^ and measures of facial matching and facial identification.^17^

**Emotion processing.** *Ekman 60 task*30 - Recognition of 60 facial expressions across six basic emotions (anger, disgust, fear, happiness, sadness, surprise) was assessed using stimuli from the Pictures of Facial Affect series.^31^ Stimuli were presented pseudorandomly for 5 seconds on a computer screen, and participants were required to select the label that best described the emotional expression. Emotion labels were present throughout testing and selection was untimed. The maximum score for this task is 60 points.

*The Awareness of Social Inference Test (TASIT)*32 - This task assesses the perception of emotions within an ecologically valid setting and consists of 24 short video clips in which an actor portrays one of six basic emotions (anger, disgust, fear, sadness, surprise, happy) as well as a Neutral, non-emotional, condition. Participants were required to view each video clip, following which a pause occurred in which participants completed the accompanying questions. A maximum of 5 points was awarded for each emotion category.

*Interpersonal Reactivity Interview (IRI)*33 - Spouses of SD patients completed the IRI as an index of the patient's present interpersonal functioning. The IRI is a 28-item questionnaire consisting of four 7-item subscales; Perspective Taking (PT; the capacity to imagine the cognitive perspective of another person), empathic concern (EC; the ability to perceive another person's emotional state), fantasy (FS; the capacity to project oneself into experiences of imaginary characters), and personal distress (PD, the tendency towards feeling anxiety in response to experiencing others in distress).

**Statistical analyses.** Data were analysed using IBM SPSS Statistics version 20. For general cognitive screening and performance on the Ekman 60 task, multivariate analyses of variance (ANOVA) were run, with Sidak post hoc comparisons used to explore group differences. Given the smaller sample size tested on the TASIT and IRI, non-parametric Kruskal-Wallis tests were run for overall group comparisons, and Mann-Whitney U tests were employed for post hoc comparisons. Finally, Spearman rank correlations, corrected for multiple comparisons (p<0.01) explored possible relationships between the experimental variables.

## RESULTS

**Demographics.** Demographic and clinical data are presented in Table 1. The groups were matched for age (F(2, 39)=0.951, p=0.395) and years in education (F(2,39)= 1.376, p=0.265). Chi-squared tests revealed that sex (χ^2^(2)=2.880, p=0.247) and handedness (χ^2^(4)=3.635, p=0.458) did not differ between the participant groups. Further, no significant differences were evident between the left and right SD cases for disease duration (i.e. the time elapsed between symptom onset and testing, F(1, 20)=1.521, p=0.232) or disease severity (CDR Sum of Boxes, F(1, 16)=0.049, p=0.828; ACE-R total score, F(1, 20)=0.181, p=0.675).

**Table 1:** Demographic and clinical characteristics of study cohorts (standard deviations in brackets).^a,b^

Demographics and cognitive tests	Left SD (n=12)	Right SD (n=10)	Controls(n=20)	F test	Post hoc test
Sex (M/F)	9:3	4:6	11:9	n/s	
Age (years)	64.9 (7.0)	68.0 (6.7)	67.7 (5.3)	n/s	n/s
Education (years)	12.9 (3.4)	11.1 (3.6)	12.8 (2.0)	n/s	n/s
Disease duration (months)	62.2 (25.9)	50.4 (17.3)	n/a	n/s	Right = Left
Handedness (R/L)	11:1	9:1	19:1	n/s	n/s
CDR sum of boxes	9.3 (4.1)	9.9 (6.3)	0 (0)	n/s	n/s
ACE-R (100)	55.9 (11.7)	58.6 (17.8)	95.0 (2.5)	**	SD < ControlsRight=Left
RCF3-min recall (36)	12.3 (9.7)	8.1 (6.0)	20.0 (4.6)	*	SD < ControlsRight = Left
Naming (30)	5.1 (2.9)	7.4 (4.3)	26.7 (2.3)	**	SD < ControlsRight = Left
Comprehension (30)	18.5 (4.6)	15.4 (6.2)	28.9 (1.50	**	SD < ControlsRight = Left
Trail Making TestPart A (s)	32.6 (7.7)	52.2 (25.4)	31.0 (8.0)	**	SD < ControlsRight < Left
Letter Fluency	8.4 (3.8)	8.3 (4.9)	41.9 (13.3)	**	SD < ControlsRight = Left
Facial Matching task (42)	38.6 (3.4)	37.7 (2.5)	39.5 (0.8)	n/s	n/s
Facial Identification task (42)	35.6 (5.2)	26.6 (3.6)	37.6 (3.0)	**	SD < ControlsRight < Left
CBI Total (%)	24.0 (15.7)	38.5 (16.7)	n/a	*	Right > Left

a Maximum score for each test in brackets where applicable.

b CDR data available for 11 left SD and 7 right SD; Trail Making test data available for 11 left SD and 8 right SD, Digits backwards available for 9 right SD, Letter fluency available for 11 left SD and 7 right SD, RCF recall available for 6 right SD, Naming and Comprehension data available for 9 right SD, Facial matching and identification discrimination task data available for 7 right SD.

* p<0.001;

** p<0.0001; R=right; L=left; n/s=non-significant; n/a=not applicable; CDR=Clinical Dementia Rating Scale; CBI=Cambridge Behavioural Inventory.

**General cognitive functioning.** Neuropsychological testing results are presented in Table 1. On the overall screening ACE-R measure, group differences were evident (F(2, 39)=65.79, p<0.0001), with both SD groups scoring significantly lower than controls (all p values <0.0001). Striking semantic processing deficits were evident on the Naming (F(2, 38)=240.86, p<0.0001) and Comprehension (F(2, 38)=47.31, p<0.0001) tasks with controls scoring significantly higher than both left and right SD cases (all p values <0.0001) and no differences between the SD subgroups (all p values >0.2). On a non-verbal test of episodic memory recall, a group effect was again observed (F(2, 35)=9.23, p=0.001) with both left (p=0.011) and right (p=0.002) SD cases showing significant impairments relative to controls, but no subgroup differences (p=0.531). Overall group differences were evident on the letter fluency task (F(2, 35)=51.35, p<0.0001) with severe fluency deficits in both SD groups relative to controls (all p values <0.0001) but no difference between the SD subgroups (p>0.9). Similarly, significant impairments were observed on the Trail Making test Part A (F(2, 35)=8.53, p=0.001), driven by difficulty exclusively in the right SD group relative to controls (p=0.001). In contrast, left SD cases scored in line with controls for Trails Part A (p=0.983), performing significantly better than their right-sided counterparts (p=0.006). Finally, on a test of facial perception, no group differences were evident (p=0.153), however, significant impairments were found on a facial identification discrimination task (F(2, 36)=21.01, p<0.0001) driven exclusively by severe deficits in the SD right group (p<0.0001) with respect to controls (left SD, p=0.417), consistent with previous reports of prosopagnosia in right SD.

Caregiver ratings of behavioural changes on the Cambridge Behavioural Inventory (CBI)^34^ revealed a significant difference between the SD subgroups (U=32.0, p=0.032) with right SD cases showing greater behavioural changes in comparison to the left SD subgroup.

In summary, left and right SD cases displayed comparable impairments in semantic processing, and episodic memory, with disproportionate speed of processing, facial identification, and behavioural disturbance evident in the right SD group.

**Figure 2:**
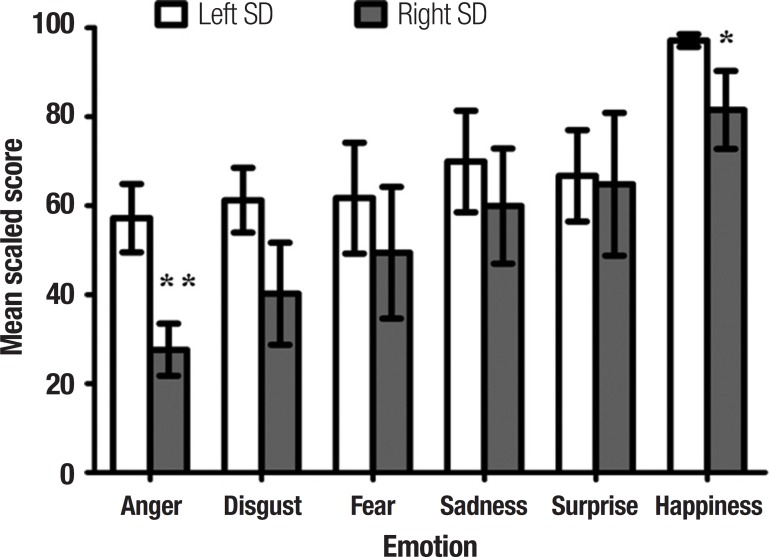
Performance of left and right SD cases on the Ekman 60 emotion recognition task. Scores for SD cases are represented as percentages of control performance. Ekman 60 data available for 9 right SD patients. Error bars depict standard error of the mean. *p<0.05; **p<0.01.

**Ekman 60 performance.** Figure 2 shows overall group performance on the Ekman 60 task. A repeated measures ANOVA revealed a significant main effect of group (F(2, 38)=28.46, p<0.0001) with both SD subgroups showing gross impairments on the Ekman 60 emotion recognition task irrespective of valence ( all p values <0.0001). No significant overall differences were evident between left and right SD subgroups (p=0.115). A main effect of valence was observed (F(5, 190)=26.54, p<0.0001), which reflected the fact that recognition of the emotion happiness was significantly higher, irrespective of group, in comparison with all other emotion categories (all p values <0.0001).

A significant group by valence interaction was found (F(10, 190)=3.02, p=0.001), which was driven by differential patterns of performance in each SD subgroup. Post hoc Sidak tests confirmed that in left SD, the recognition of all negative emotions was markedly disrupted relative to controls (anger, p<0.0001; disgust, p<0.0001; fear, p=0.028; sadness, p=0.020). Recognition of surprise was also significantly impaired in this group (p=0.025). In contrast, recognition of happiness was intact in the left SD cohort with respect to controls (p=0.900).

For right SD cases, striking impairments for all basic emotions were observed (anger, p<0.0001; disgust, p<0.0001; fear, p=0.006; sadness, p=0.004; surprise, p=0.025) including the recognition of happiness (p=0.002) relative to controls. Further post hoc analyses confirmed that right SD cases performed significantly poorer than left SD cases for the recognition of anger (p=0.005) and happiness (p=0.021), with no other significant differences detected between the subgroups (all p values >0.1).

To investigate the contribution of perceptual processes on facial emotion recognition, an analysis of covariance (ANCOVA) was run with performance on the facial matching task included as a covariate. The main effect of diagnosis persisted (F(2, 35)=19.63, p<0.0001), with both left and right SD cases displaying significant overall impairments with respect to controls (all p values < .0001). A significant valence by group interaction was also evident (F(10, 175)=3.22, p=0.001) which was driven by severe impairments in the recognition of specific emotions in each SD subgroup. Left SD cases continued to show marked deficits relative to controls for the recognition of anger (p<0.0001), disgust (p<0.0001), sadness (p=0.023) and surprise (p=0.016) with intact recognition of fear (p=0.067) and happiness (p=0.985). In contrast, right SD cases' deficits remained present for the recognition of anger (p<0.0001), disgust (p<0.0001), fear (p=0.044), sadness (p=0.022), and happiness (p=0.027), but recognition of surprise was intact (p=0.594). Looking between the SD subgroups, right SD cases scored significantly lower than left SD cases for the recognition of anger (p=0.034) with no other differences between subgroups found (all p values >0.05).

A separate ANCOVA was also carried out using naming performance as a covariate, to control for the possible influence of semantic processing on the labelling of emotions. The main effect of diagnosis persisted (F(2, 37)=5.610, p=0.007); however, post hoc tests revealed that SD groups did not differ significantly from controls for overall emotion recognition performance (Right SD, p=0.957; Left SD, p=0.279). Interestingly, right SD cases continued to score significantly lower than left SD cases (p=0.006), for the recognition of anger (p=0.001), disgust (p=0.012), and happiness (p=0.008).

**The Awareness of Social Inference Test (TASIT).** A Kruskal Wallis test revealed significant group differences for the recognition of positive (χ^2^(2)=16.26, p<0.0001), and negative (χ^2^(2)=26.73, p<0.0001) emotions on the

TASIT. Mann Whitney U tests demonstrated that left SD cases were significantly impaired for positive (U=37.5, p=0.001) and negative (U=3.5, p<0.0001) emotions relative to controls. Likewise, right SD cases showed significant impairments across positive (U=5.0, p=0.001) and negative (U=0.000, p<0.0001) emotions on the TASIT with respect to controls. No significant differences were evident between the SD subgroups (positive, U=22.0, p=0.221; negative, U=16.5, p=0.080).

Looking at the subscales of the TASIT, a Kruskal Wallis test revealed overall group differences for recognition of the following emotions; anger (χ^2^(2)=15.61, p<0.0001), fear (χ^2^(2)=15.94, p<0.0001), disgust (χ^2^(2)=19.19, p<0.0001), and surprise (χ^2^(2)=19.82, p<0.0001) with the suggestion of a group difference for sadness (χ^2^(2)=5.66, p=0.059) (Table 2). Mann Whitney U tests confirmed that left SD cases were significantly impaired with respect to controls for recognition of anger (U=34.0, p<0.0001), fear (U=59.0, p=0.017), disgust (U=27.5, p<0.0001), surprise (U=38.5, p=0.001), and mild deficits observed for recognition of happiness (U=70.0, p=0.053). Similarly, right SD cases showed significant impairments relative to controls for the recognition of surprise (U=2.5, p<0.0001), anger (U=11.5, p=0.006), fear (U=1.5, p<0.0001) and disgust (U=3.5, p<0.0001), but preserved recognition of happiness (U=38.0, p=0.447). No significant subgroup differences were evident between the SD subgroups for any of the TASIT emotion categories (anger, U=29.0, p=0.479; disgust, U=26.0, p=0.360; fear, U=16.5, p=0.080; sadness, U=22.5, p=0.221; happiness, U=26.0, p=0.360; surprise, U=23.5, p=0.253).

**Table 2:** Performance of left and right SD cases and controls on subscales of the TASIT emotion recognition task.

TASIT A subscale	Left SD	Right SD	Controls	Group difference
Anger	1.8 (1.1)	2.0 (0.7)	3.3 (0.7)	*
Disgust	1.4 (1.3)	1.0 (1.0)	3.4 (0.8)	*
Fear	2.9 (2.4)	1.4 (1.1)	3.8 (0.4)	*
Sadness	2.3 (0.9)	2.0 (1.2)	2.9 (0.8)	0.059
Surprise	2.2 (1.5)	1.8 (0.8)	3.8 (0.5)	*
Happy	3.2 (0.7)	3.4 (0.9)	3.8 (0.4)	n/s
Neutral	1.7 (1.1)	1.4 (1.5)	2.5 (0.8)	n/s

Standard deviations are shown in brackets. TASIT data available for 5 right SD and 12 left SD patients.

* p<0.0001 based on non-parametric Kruskal Wallis test.

**Figure 3:**
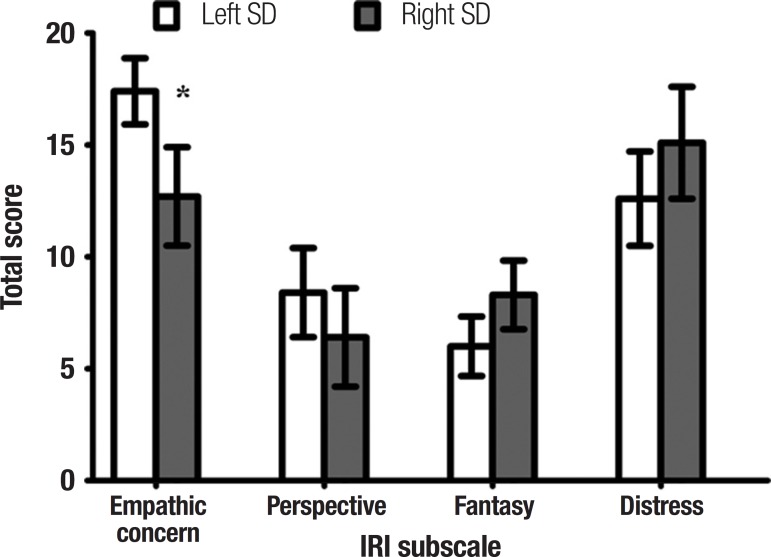
Caregiver ratings of interpersonal changes on the Interpersonal Reactivity Interview (IRI) in left and right SD cases. IRI data available for 11 left SD and 9 right SD cases. Error bars depict standard error of the mean. *p<0.05.

**Caregiver ratings of interpersonal changes.** Investigating the SD subgroups on carer rated measures of interpersonal reactivity, Mann-Whitney U tests failed to reveal significant group differences for caregiver ratings of perspective taking (U=36.5, p=0.165), fantasy (U=33.0, p=0.178), and personal distress (U=42.0, p=0.302) on the IRI. Right SD cases, however, were rated as demonstrating less empathic concern relative to left SD cases (U=27.0, p=0.047) (Figure 3).

**Correlations between experimental measures.** Spearman rank correlations for the overall SD cohort (n=22), adjusted for multiple comparisons at p<0.01 level, are presented in Table 3. Performance on the Ekman 60 task was significantly related to global cognitive functioning, and total TASIT performance, with higher performance on the Ekman 60 associated with lower incidences of behavioural change on the CBI. Similarly, total TASIT performance was significantly inversely related to degree of behavioural change on the CBI. Further, ratings of empathic concern on the IRI inversely correlated with behavioural changes on the CBI.

**Table 3:** Spearman rank correlations showing relationships between experimental measures in the combined SD groups (n=22).

	ACE-R Total	TASIT Total	CBI Total
Ekman 60	0.546*	0.874**	-0.651**
TASIT	0.328	1.000	-0.606*
IRI Empathy	0.100	0.560	-0.596*

Correlations were adjusted for multiple comparisons using a corrected alpha level of 0.01;

* p<0.005;

** p<0.001

## DISCUSSION

The role of the anterior temporal lobes in mediating successful social interactions remains a source of debate within neuropsychology. Using two well-characterised groups of patients with SD, in which the predominant burden of brain atrophy was lateralised to either the left or right anterior temporal lobes, we found marked deficits in the ability to recognise basic emotions, irrespective of lateralisation of atrophy. Importantly, differences between the two SD groups, however, were evident for the recognition of specific emotions and the emotional aspects of empathy (i.e., empathic concern), pointing towards the importance of the right anterior lobe for discrete aspects of interpersonal functioning.

The finding of marked alterations in the recognition of basic facial emotions in SD resonates with previous reports in the literature, in particular for the recognition of negative emotions.^16,17^ Left SD cases displayed marked difficulties in the recognition of all negative emotions on the Ekman 60, as well as surprise, deficits which were not related to naming or general semantic processing capacity. In contrast, right SD cases displayed profound deficits in the recognition of all basic facial emotions, scoring significantly poorer than the left SD group for the recognition of anger and happiness. Importantly, while Ekman 60 performance correlated significantly with facial identity discrimination in right SD, our covariate analysis suggests that general face processing disturbances, and semantic naming impairments, do not fully account for the marked emotion recognition deficits found in this group. Degeneration of the right anterior temporal lobe appears critical in the genesis of global emotion processing difficulties in right SD. The right amygdala is the most likely candidate driving such disruption, a structure which has previously been implicated in disruption of negative emotion recognition,^35^ and behavioural changes including disinhibition and depression in SD.^36^ Findings from the Ekman 60 task were largely replicated on the TASIT, with a number of important exceptions. Both SD subgroups displayed significant impairments for the recognition of negative emotions, as well as surprise. Subgroup analyses, however, uncovered a relatively spared capacity to recognise happiness in the right SD group. Unlike on the Ekman 60, differences between SD groups were not evident on the TASIT, a finding that likely relates to the provision of rich contextual information on this task. Right SD patients may benefit from the additional multimodal information provided on the TASIT, such as tone, prosody, and gesture, thus reducing differences between SD subgroups. These findings lend support to the proposal that the right temporal lobe is specialised for the processing of facial stimuli.^37^

Given the evidence pointing to the importance of right temporal structures in facilitating interpersonal behaviours including empathy,^8,20,38-39^ the disproportionate deficits found on the empathic concern subscale of the IRI in the right SD cases are not surprising. Patients with right predominant SD are typically held to show marked reduction of interpersonal functioning with reports of "cold-heartedness" and loss of warmth. The inability to share emotional experiences in this manner, in turn, likely impacts on the capacity for perspective taking, and the suppression of one's own viewpoint, particularly as the pathological process begins to encroach into adjacent frontal regions.^20^ The status of complex self-projective social cognitive functions in SD remains poorly understood.^40^ Recent evidence, however, points towards striking deficits in theory of mind in left SD cases.^41^ Whether SD patients with predominantly right-sided pathology show theory of mind deficits of a greater magnitude than left SD cases remains to be established, but this seems a plausible assumption.

To our knowledge, this study represents the first concerted effort to investigate differences in interpersonal functioning in left versus right SD. Given the size of our sample, further investigations in larger independent samples will be important to confirm our findings. Another important consideration relates to the disease staging of our SD participants. Over time, the pathological process in SD spreads from one anterior lobe to the other, resulting in bilateral insult to the amygdalae, as well as encroachment of atrophy into ventromedial prefrontal areas.^42,43^ With disease progression, symptoms undetected at baseline become evident, resulting in a mixed clinical presentation.^13,42^ Finally, the binary classification into left or right SD obscures the fact that a degree of bilateral atrophy is often present in these patients.^2,4^ Future studies incorporating automated neuroimaging analyses, such as voxel-based morphometry, to quantify the extent of left and right anterior lobe atrophy in each subgroup are thus warranted.

In summary, we have demonstrated a considerable overlap in the extent to which socioemotional processes are disrupted in SD cases with predominant left or right temporal lobe atrophy. Despite these common features, however, right SD cases show disproportionate deficits in the recognition of basic emotions, and in their capacity for empathic concern. Future studies investigating associations between regional brain integrity and performance on emotion processing tasks will provide valuable information regarding the relative contribution of left versus right anterior temporal structures to socioemotional functioning in SD.
